# BiClamp® vessel-sealing device for open hepatic resection of malignant and benign liver tumours: a single-institution experience

**DOI:** 10.1186/s12885-017-3513-0

**Published:** 2017-08-22

**Authors:** Yi-jun Zhao, Da-chen Zhou, Fu-bao Liu, Hong-chuan Zhao, Guo-bin Wang, Xiao-ping Geng

**Affiliations:** 1 Department of Hepatopancreatobiliary Surgery II, the First Affiliated Hospital of Anhui Medical University, No. 218 Jixi Road, Hefei, Anhui Province 230022 China; 2 Department of Surgery, The Second Affiliated Hospital of Anhui Medical University, No. 678 Furong Road, Hefei, Anhui Province 230601 China

**Keywords:** Hepatectomy, Intraoperative blood loss, BiClamp®, Vessel sealing

## Abstract

**Background:**

Intraoperative blood loss during hepatectomy worsens prognosis, and various tools have been used to improve perioperative safety and feasibility. We aimed to retrospectively evaluate the feasibility and safety of the BiClamp® device for open liver resection.

**Methods:**

We included 84 patients undergoing liver resection from a single centre, with all patients operated by the same surgical group. All hepatectomies were performed using BiClamp® (Erbe Elektromedizin GmbH, Tubingen, Germany), an electrosurgical device that simultaneously transects liver parenchyma and seals vessels <7 mm in diameter. We collected data on intraoperative blood loss, resection time, and perioperative complications, comparing cirrhotic and non-cirrhotic patients.

**Results:**

The 84 patients enrolled in this study included 56 cirrhotic and 28 non-cirrhotic patients. All patients underwent hepatectomy (30 major and 54 minor hepatectomies) using the BiClamp®, exclusively, and 54 patients required inflow occlusion (Pringle manoeuvre). Overall intraoperative blood loss (mean ± standard deviation) was 523.5 ± 558.6 ml, liver parenchymal transection time was 36.3 ± 16.5 min (range, 13-80 min), and the mean parenchymal transection speed was 3.0 ± 1.9 cm^2^/min. Twelve patients received perioperative blood transfusion. The cost of BiClamp® for each patient was 800 RMB (approximately 109€). There were no deaths, and the morbidity rate was 25%. The mean (standard deviation) hospital stay was 9.3 (2.3) days. Comparisons between cirrhotic and non-cirrhotic patients revealed no difference in blood loss (491.0 ± 535.7 ml vs 588.8 ± 617.5 ml, *P* = 0.598), liver parenchymal transection time (34.1 ± 14.8 min vs 40.9 ± 19.2 min, *P* = 0.208), mean parenchymal transection speed (3.3 ± 2.1 cm^2^/min vs 2.5 ± 1.3 cm^2^/min, *P* = 0.217), and operative morbidity (28.6% vs 14.3%, *P* = 0.147).

**Conclusions:**

The reusable BiClamp® vessel-sealing device allows for safe and feasible major and minor hepatectomy, even in patients with cirrhotic liver.

**Trial registration:**

This trial was retrospectively registered and the detail information was as followed. Registration number: ChiCTR-ORC-17011873 (Chinese Clinical Trial Registry). Registration Date: 2017-07-05.

**Electronic supplementary material:**

The online version of this article (doi:10.1186/s12885-017-3513-0) contains supplementary material, which is available to authorized users.

## Background

Liver resection is a demanding procedure that is often the only chance of a cure for many patients [1, 2]. Intraoperative blood loss during hepatectomy is a significant factor in determining postoperative morbidity and mortality [3], and several surgical techniques have been recommended to reduce blood loss. Inflow occlusion (Pringle manoeuvre) and low central venous pressure anaesthesia are proven effective methods to minimize haemorrhage during parenchymal transection [4, 5]. Since the first introduction of the clamp-crushing technique in the 1970s [6], surgical strategies to reduce blood loss in patients undergoing hepatectomy have focused mainly on technical innovations in liver parenchymal transection, including an ultrasonic dissector (CUSA®, Tyco Healthcare, Mansfield, MA, USA), the Waterjet® (Erbe Elektromedizin GmbH, Tubingen, Germany), a dissecting sealer (TissueLink®, Dover, NH, USA), and others. However, randomised controlled trials have shown that the clamp-crushing technique remains the reference technique for liver parenchymal transection compared with alternative transection techniques [7].

BiClamp® (Erbe Elektromedizin GmbH) was designed as a reusable bipolar vessel-sealing device that successfully ligates 2–7 mm arteries and veins [8]. The shape of BiClamp® is similar to a typical clamp; therefore, it can be used in liver transection similar to the clamp-crushing technique, and the device’s coagulation function may address limitations in the clamp-crushing technique. Uchiyama and colleagues used BiClamp® in laparoscopic partial hepatectomy [9]. The median tumour diameter of the nine patients in their study was 1.5 cm, and results confirmed the safety and efficacy of BiClamp® in laparoscopic partial hepatectomy. In another study, BiClamp® was added for liver parenchymal transection with the CUSA device [10]. The authors found lower intraoperative blood loss in the BiClamp® group compared with the group receiving CUSA combined with bipolar electrocautery.

To our knowledge, there are no previous publications evaluating the use of BiClamp® for open liver resection, especially in patients with cirrhosis. We performed this respective study to evaluate the feasibility and safety of this surgical device for open liver parenchymal transection [11].

## Methods

### Previous experience

We have used the BiClamp® for liver resection since 2007, and more than 200 patients underwent hepatectomy in our institution before the current trial. For patient safety reasons, the clamp-crushing technique and inflow occlusion (Pringle manoeuvre) were necessary when first using the BiClamp® in our institution. However, after obtaining acceptable operative complication and blood transfusion rates, we realized that liver resection can be performed using the BiClamp® exclusively. The overall rate of inflow occlusion decreased during this study’s duration because inflow occlusion is unnecessary when the intrahepatic duct structure is clearly distinguishable, which decreases ischemia reperfusion injury caused by hepatic ischemia.

### Experimental design

We enrolled 84 consecutive patients between July 2010 and July 2012, and all patients were operated by the same surgical group. Eligibility criteria included patients with benign and malignant tumours and acceptable liver function (Child-Pugh score A), and acceptable status to undergo the operation. Patients undergoing local partial hepatectomy for less than one segment were excluded from the study.

### Surgical procedure

Intraoperative ultrasound was used to define the tumour location relative to the major vascular and biliary structures. The power supply for the BiClamp® was fixed at level 4, and the effect was at set at 100. Inflow occlusion was performed only when blood loss exceeded the expected amount.

**Fig. 1:**
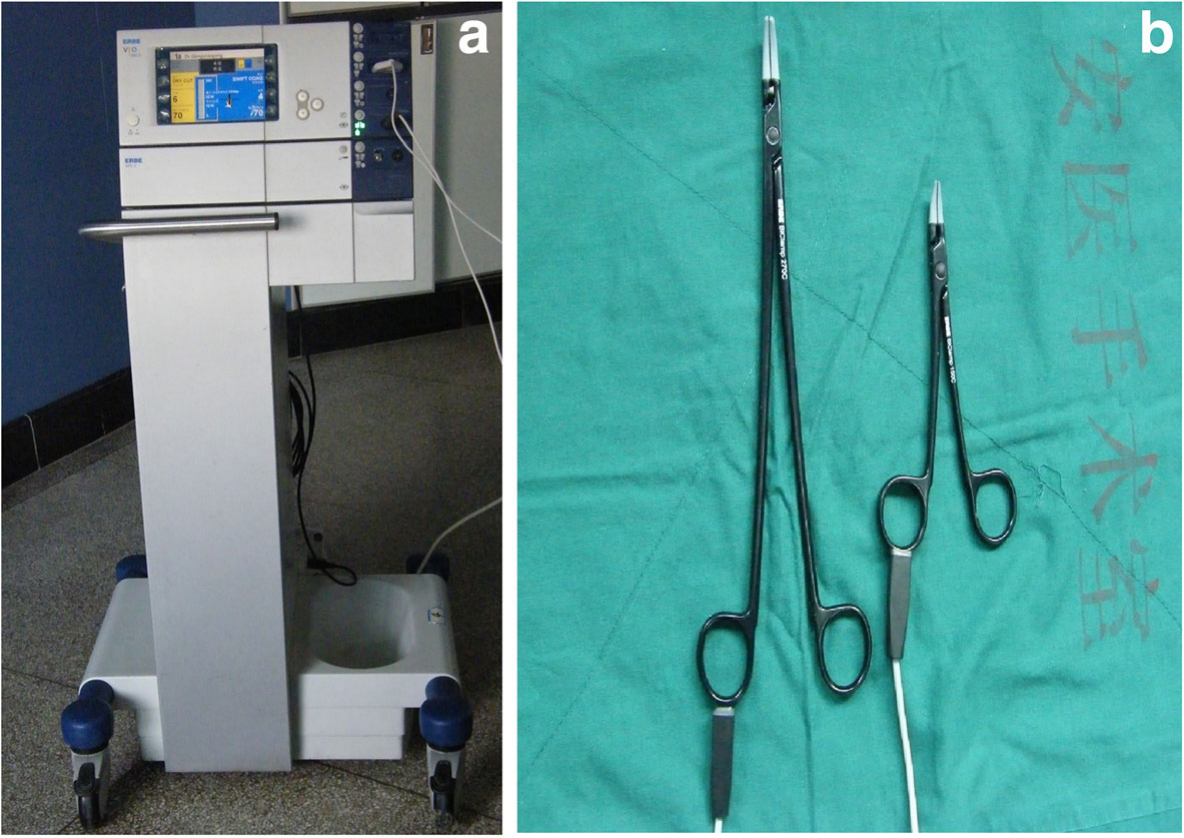
**a** The ERBE VIO 300D energy platform for the reusable BiClamp® device, and (**b**) the BiClamp® device showing the comparative size of the normal and small clamping forceps

**Fig. 2:**
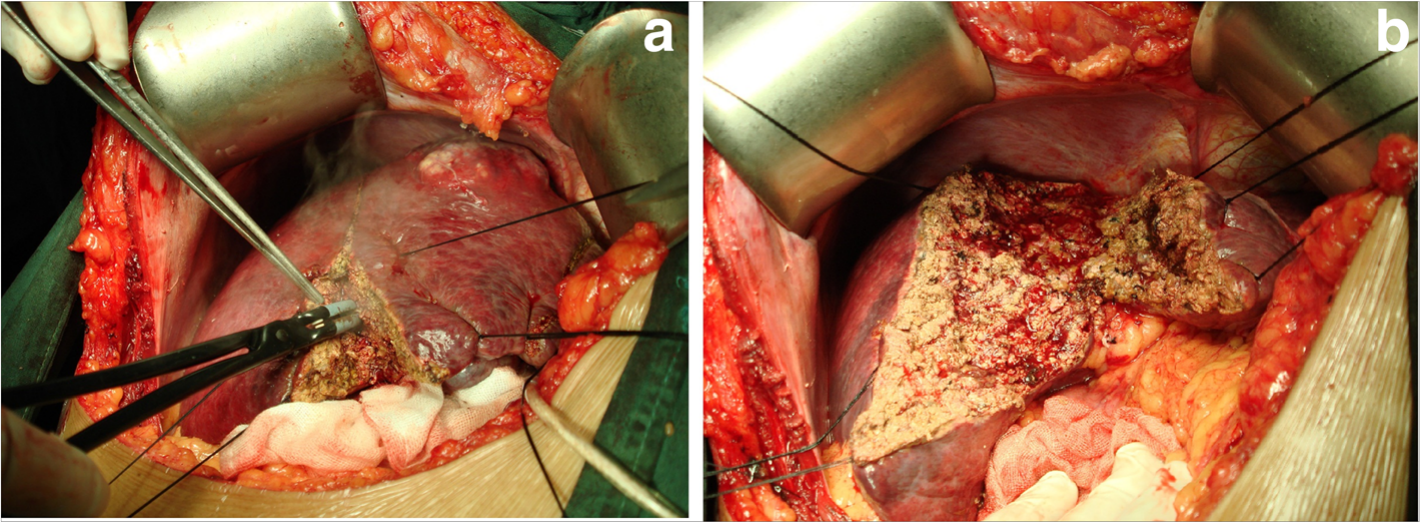
**a** Photo showing the hepatic parenchyma being transected by the BiClamp®, and (**b**) the bloodless liver cut surface has no carbonization, which often appears as black burning

The BiClamp® has two blades that can crush liver parenchyma like a clamp (Fig. 1), and the device’s coagulation function seals intrahepatic vessels simultaneously (Fig. 2). Larger intrahepatic structures can be isolated by the BiClamp®; vessels can then be divided by an electrotome or scissors followed by standard ligation or ligation with titanium clips (see Additional file 1: Video S1).

All resections in our study were performed with low central venous pressure (0–5 mmHg), and prophylactic postoperative drainage was performed routinely. Major hepatectomy was defined as liver resection involving ≥ three segments, and minor hepatectomy was defined as resection of < three segments.

### Outcome measures

Each patient was followed for at least 3 months and the safety of the BiClamp® was evaluated based on postoperative morbidity and mortality. The feasibility of the BiClamp® was evaluated by the speed of liver parenchymal transection and by blood loss. Resection time was defined as the duration between the beginning and the end of parenchymal transection. Following transection, blood loss volume was estimated by the weight of the suction fluid and blood-soaked swabs after subtracting the lavage fluid volume and the weight of any dry swabs that were used during transection using the formula 1 g = 1 ml of blood.

A sheet of sterile paper was placed against the liver cut surface immediately following liver resection to duplicate the liver cut-surface area. The outline of the cut surface on the sterile paper was copied to a sheet of dry 120 g/m^2^ paper, postoperatively. The liver cut-surface area was then calculated using the 120 g/m^2^ density of the second paper. The transection area was expressed as cm^2^, and the speed of transection was expressed as the transection area divided by the transection time (cm^2^/min). Complications were recorded based on severity according to the classification system [12]. Mortality was defined as death within 30 days of hepatectomy. Bile leakage was defined as a total bilirubin level ≥ 86 μmol/L in the drained fluid [13], and the indications for blood transfusion included blood loss >1500 ml or a haemoglobin level < 70 g/L.

### Statistical analysis

Results are shown as mean ± standard deviation (SD). Fischer’s exact, χ^2^, and independent samples t-tests were used when needed. The difference was considered significant if *P* < 0.05. All statistical calculations were performed using SPSS 17.0 (SPSS, Inc., Chicago, IL).

## Results

The following neoplasms occurred in the 84 patients in this study: hepatocellular carcinoma (56 cases), cholangiocarcinoma (6 cases), colorectal liver metastases (6 cases), and haemangioma (16 cases). There were 74 first, 6 s, and 4 third hepatectomies. Patients’ clinical details are shown in Table 1.

**Table 1:** Patient demographics and tumour characteristics

Characteristic	Finding
No. of patients	84
Gender, No, (F/M)	18/66
Age, Mean ± standard deviation (SD), (Y)	51.8 ± 11.3
Number of patients with cirrhosis	56
Hepatocellular carcinoma	56
Cholangiocarcinoma	6
Colorectal Metastases	6
Haemangioma	16
Tumour Size, Mean ± SD, (mm)	72 ± 32
No. of tumours, Mean ± SD	1.6 ± 1.3

Thirty patients underwent major hepatectomies and 54 patients underwent minor hepatectomies. The intraoperative transection-related features and surgical outcomes are shown in Table 2. The overall operative time was 168.9 ± 44.7 min (range, 100–300 min), and the parenchymal transection time was 36.3 ± 16.5 min (range, 13–80 min). The liver cut-surface area measurement was 95.1 ± 43.9 cm^2^, parenchymal transection time per square centimetre was 0.43 ± 0.23 min, and the speed of parenchymal transection was 3.0 ± 1.9 cm^2^/min. The overall intraoperative blood loss was 523.5 ± 558.6 ml (range, 55.0–2474.1 ml), and the mean blood loss volume per square centimetre was 6.2 ± 7.6 ml (range, 0.6–39.8 ml). Fifty-four patients required hepatic inflow occlusion (Pringle manoeuvre) during liver resection, but only 12 patients required intraoperative blood transfusion. No patients developed grounding pad skin burn, myocardial infarction, or cardiac arrhythmia during or after the operation, and there were no deaths within 30 days postoperatively. The cost of BiClamp® for each patient was 800 RMB (approximately 109€).

**Table 2:** Intraoperative transection-related features and surgical outcomes (*n* = 84)

Features	Finding
Operative time (minutes)	168.9 ± 44.7 (168.9 ± 44.7)
Parenchymal transaction time (minutes)	36.3 ± 16.5 (13-80)
Liver cut suface area (cm^2^)	95.1 ± 43.9 (31.82 ~ 202.43)
Mean transection speed (cm^2^/min)	3.0 ± 1.9
Total blood loss (ml)	523.5 ± 558.6(55.0 ~ 2474.1)
Mean blood loss (mL/cm^2^)	6.2 ± 7.6 (0.6 ~ 39.8)
No. of transfused patients	12 (14.3%)
No. of patients with Pringle (%)	54 (64.3%)
Overall operative morbidity	20 (25%)
Operative mortality	0 (0%)
Inhospital (days)	9.3 ± 2.7
Expense of BiClamp for each patient	800 RMB (approximately 109€)

Fifteen patients suffered grade 1–2 postoperative complications according to the new classification [12] with 13 patients developing right pleural effusion, which was diagnosed on upper abdominal CT. All 13 patients recovered smoothly without additional treatment. One patient developed biliary leak that was diagnosed by the total bilirubin level in the abdominal drainage fluid [13]; this resolved after 2 weeks of postoperative drainage. One patient suffered transient liver dysfunction with pleural effusion and ascites that resolved with albumin therapy and diuresis. No patients developed postoperative bleeding and none required reoperation. The mean postoperative hospital stay was 9.3 ± 2.3 days (range, 5–18 days). No readmissions occurred within the 3-month follow-up.

The surgical characteristics for patients in the cirrhotic and non-cirrhotic groups are compared in Table 3 *.* Other than the significantly higher proportion of males in the cirrhotic group compared with the non-cirrhotic group, there were no differences between the two groups for: operation time (166.4 ± 39.2 min vs 174.0 ± 55.5 min, *P* = 0.611), parenchymal transection time (34.1 ± 14.8 min vs 40.9 ± 19.2 min, *P* = 0.208), liver cut-surface area (98.4 ± 48.1 cm^2^ vs 88.7 ± 35.0 cm^2^, *P* = 0.508), mean transection speed (3.3 ± 2.1 cm^2^/min vs 2.5 ± 1.3 cm^2^/min, *P* = 0.217), blood loss (491.0 ± 535.7 ml vs 588.8 ± 617.5 ml, *P* = 0.598), Mean blood loss (mL/cm2) (5.5 ± 6.1 VS 7.6 ± 10.1, *P* = 0.406), number of patients requiring transfusion (6/56 vs 6/28, *P* = 0.321), patients receiving the Pringle manoeuvre (34/56 vs 20/28, *P* = 0.469), complications (16/56 vs 4/28, *P* = 0.147), or length of hospital stay (9.5 ± 3.1 days vs 9.1 ± 2.1 days, *P* = 0.635).

**Table 3:** Comparison of surgical results between patients with and without cirrhosis

Features	Cirrhosis (*n* = 56)	Non-cirrhosis (*n* = 28)	*P* value
Gender, No, (F/M)	3/53	15/13	0.000^a^
Age, (Y)	50.4 ± 12.3	54.9 ± 8.7	0.228
Operative time (minutes)	166.4 ± 39.2	174.0 ± 55.5	0.611
Parenchymal transaction time (minutes)	34.1 ± 14.8	40.9 ± 19.2	0.208
Liver cut surface area (cm^2^)	98.4 ± 48.1	88.7 ± 35.0	0.508
Mean transection speed (cm^2^/min)	3.3 ± 2.1	2.5 ± 1.3	0.217
Total blood loss (ml)	491.0 ± 535.7	588.8 ± 617.5	0.598
Mean blood loss (mL/cm^2^)	5.5 ± 6.1	7.6 ± 10.1	0.406
No. of transfused patients (0%)	6 (10.7%)	6 (21.4%)	0.321^b^
No. of patients with Pringle (%)	34 (42.9%)	20 (71.4%)	0.469^b^
Operative morbidity (%)	16 (28.6%)	4 (14.3%)	0.147^a^
Inhospital (days)	9.5 ± 3.1	9.1 ± 2.1	0.635

Comparisons of mean ± SD used independent samples t-tests; ^a^Fisher’s exact; ^b^χ^2^

## Discussion

BiClamp® is an innovative bipolar coagulation system with adjustable current modulation and intelligent self-control, which transforms electrical energy into heat, thus attaining an ideal energy-based vessel seal. Vessels with a diameter ≤ 7 mm can be successfully sealed by BiClamp alone [9]. BiClamp® is a reusable, cost-effective instrument.

BiClamp® has been widely used in thyroidectomies, hysterectomies, and pulmonary lobectomies, with proven efficacy and safety [14–16]. However, experience using the BiClamp® in open liver resections is limited. BiClamp® has two blades that simultaneously crush liver parenchyma like a clamp and seal intrahepatic vessels via the coagulation function. Theoretically, BiClamp®‘s vessel-sealing function could be more effective in liver parenchymal transection than clamp crushing.

In Uchiyama and colleagues’ study [10], BiClamp® was applied to laparoscopic hepatectomies, and the estimated blood loss was as low as 417 ml with all patients recovering smoothly without complications. In another study [10] in which BiClamp® was used in addition to CUSA, the median blood loss was 345 mL in the BiClamp® group, which was less than that in the group receiving CUSA combined with bipolar electrocautery. In our patients, the mean tumour diameter was 7.2 cm, and 30 patients underwent major hepatectomies including 10 anatomical hemi-hepatectomies.

Our results showed that some complicated hepatectomies can be performed safely and efficiently using BiClamp® alone, even in some cirrhotic patients. We encountered no severe postoperative morbidity and mortality according to the classification of complications by severity [12].

We found that using BiClamp® was similar to the clamp-crushing technique, and that coagulation helped with hemostasis of the liver cut surface. Intrahepatic structures are coagulated during liver parenchymal transection without transecting the vessels, using BiClamp®. As seen in the supplemental video, titanium clips were used to ligate the small vessels before transection, and 4-0 Prolene (Ethicon, Somerville, NJ, USA) was used to ligate the major intrahepatic vasculature when necessary.

Another useful feature of BiClamp® in liver parenchymal transection is that the saline lavage following coagulation prevents liver tissue from adhering to the BiClamp® blades, which also prevents carbonization of the liver cut surface. Using BiClamp® is easy; however, liver parenchymal transection must proceed slowly while activating the BiClamp®, which is better for small vessel coagulation.

Liver parenchymal transection speed and blood loss were the most important parameters when we evaluated the feasibility of BiClamp® for hepatectomy. Previous reports indicated a clamp-crushing speed of 0.89–3.9 cm^2^/min, and a mean blood loss per square centimetre of 1.5–7.0 ml [17–19]. Our results indicated a parenchymal transection speed of 3.0 ± 1.9 cm^2^/min and a mean blood loss per square centimetre of 6.2 ± 7.6 ml, similar results to previous studies.

Hepatic cirrhosis may be an adverse factor leading to increased bleeding [20]. Our results showed no difference in intraoperative blood loss, transection speed, proportion of hepatic inflow occlusion, or need for transfusion between the cirrhosis and non-cirrhosis groups using BiClamp®. The post-operative complication incidence in our cirrhosis group was higher than that in the non-cirrhosis group but with no significant difference, suggesting that BiClamp® may be more advantageous for hepatectomy in patients with cirrhosis.

Another advantage of BiClamp® was to reduce the cost for patients due to its reusable. Being calculated by deprecition and disinfection cost of instruments, each patient should only pay 800RMB (approximately 109€) for BiClamp®, while the cost of CUSA [17] and Ligasure [21] was reported 661€ and 447.2 ± 58.9€, respectively.

The limitations of this study include the retrospective, single-centre design, the small sample size, and the short-term follow-up. Prospective, randomised control trials are required to confirm our results; therefore, we designed a prospective randomised trial to compare BiClamp® with clamp-crushing methods in open liver resection (NCT02197481) [22].

## Conclusions

Overall, using the reusable BiClamp® vessel-sealing device assures hepatectomy safety and effectiveness, even in patients with cirrhotic liver.

## Additional file

Additional file 1: Video S1. Open liver resection by BiClamp®. (MP4 36943 kb)

## Abbreviations

F: Female
M: Male
SD: Standard deviation
